# Preoperative or Perioperative Docetaxel, Oxaliplatin, and Capecitabine (GASTRODOC Regimen) in Patients with Locally-Advanced Resectable Gastric Cancer: A Randomized Phase-II Trial

**DOI:** 10.3390/cancers12102790

**Published:** 2020-09-29

**Authors:** Manlio Monti, Paolo Morgagni, Oriana Nanni, Massimo Framarini, Luca Saragoni, Daniele Marrelli, Franco Roviello, Roberto Petrioli, Uberto Fumagalli Romario, Lorenza Rimassa, Silvia Bozzarelli, Annibale Donini, Luigina Graziosi, Verena De Angelis, Giovanni De Manzoni, Maria Bencivenga, Valentina Mengardo, Emilio Parma, Carlo Milandri, Gianni Mura, Alessandra Signorini, Gianluca Baiocchi, Sarah Molfino, Giovanni Sgroi, Francesca Steccanella, Stefano Rausei, Ilaria Proserpio, Jacopo Viganò, Silvia Brugnatelli, Andrea Rinnovati, Stefano Santi, Giorgio Ercolani, Flavia Foca, Linda Valmorri, Dino Amadori, Giovanni Luca Frassineti

**Affiliations:** 1 Department of Medical Oncology, Istituto Scientifico Romagnolo per lo Studio e la Cura dei Tumori (IRST) IRCCS, 47014 Meldola, Italy; dino.amadori@irst.emr.it (D.A.); luca.frassineti@irst.emr.it (G.L.F.); 2 Department of General Surgery, Morgagni-Pierantoni Hospital, 47121 Forlì, Italy; paolo.morgagni@auslromagna.it (P.M.); massimo.framarini@auslromagna.it (M.F.); giorgio.ercolani@auslromagna.it (G.E.); 3 Unit of Biostatistics and Clinical Trials, Istituto Scientifico Romagnolo per lo Studio e la Cura dei Tumori (IRST) IRCCS, 47014 Meldola, Italy; oriana.nanni@irst.emr.it (O.N.); flavia.foca@irst.emr.it (F.F.); linda.valmorri@irst.emr.it (L.V.); 4 Pathology Unit, Morgagni-Pierantoni Hospital, 47121 Forlì, Italy; luca.saragoni@auslromagna.it; 5 Unit of Surgical Oncology, Department of Medicine, Surgery and Neurosciences, University of Siena, 53100 Siena, Italy; daniele.marrelli@ao-siena.toscana.it (D.M.); franco.roviello@unisi.it (F.R.); 6 Unit of Medical Oncology, Department of Medicine, Surgery and Neurosciences, University of Siena, 53100 Siena, Italy; r.petrioli@ao-siena.toscana.it; 7 European Institute of Oncology IRCCS, 20141 Milan, Italy; ubertofumagalliromario@gmail.com; 8 Medical Oncology and Hematology Unit, Humanitas Cancer Center, Humanitas Clinical and Research Center-IRCCS, 20089 Rozzano (Milan), Italy; lorenza.rimassa@hunimed.eu (L.R.); silvia.bozzarelli@cancercenter.humanitas.it (S.B.); 9 Department of Biomedical Sciences, Humanitas University, 20090 Pieve Emanuele (Milan), Italy; 10 General and Emergency Surgery, Santa Maria della Misericordia Hospital, University of Perugia, 06129 Perugia, Italy; annibale.donini@unipg.it (A.D.); luiginagraziosi@yahoo.it (L.G.); 11 Clinical Oncology, Santa Maria della Misericordia Hospital, University of Perugia, 06129 Perugia, Italy; verdeang@gmail.com; 12 General and Upper GI Surgery Division, University of Verona, 37129 Verona, Italy; giovanni.demanzoni@univr.it (G.D.M.); mariabenci@hotmail.it (M.B.); valentina.mengardo@gmail.com (V.M.); 13 Department of Surgery, San Giuseppe Hospital, 50053 Empoli, Italy; emilio.parma@uslcentro.toscana.it; 14 Department of Oncology, San Giuseppe Hospital, 50053 Empoli, Italy; carlo.milandri@uslcentro.toscana.it; 15 Department of General Surgery, San Donato Hospital, 52100 Arezzo, Italy; gianmura@gmail.com; 16 Department of Oncology, Valdarno Hospital, 52025 Montevarchi, Italy; signo.a@libero.it; 17 Department of Clinical and Experimental Sciences, Surgical Clinic, University of Brescia, 25121 Brescia, Italy; gianluca.baiocchi@unibs.it (G.B.); sarahmolfino@gmail.com (S.M.); 18 Surgical Oncology Unit, Surgery Department, ASST Bergamo Ovest, 24047 Treviglio, Italy; giosgroi@gmail.com (G.S.); fra.steccanella@gmail.com (F.S.); 19 First Division of Surgery, Senology Research Center, Department of Surgical and Morphological Sciences, University of Insubria, 21100 Varese, Italy; stefano.rausei@gmail.com; 20 Medical Oncology, Ospedale di Circolo e Fondazione Macchi, 21100 Varese, Italy; ilaria.proserpio@ospedale.varese.it; 21 General Surgery, IRCCS San Matteo, 27100 Pavia, Italy; j.vigano@smatteo.pv.it; 22 Medical Oncology Unit, IRCCS San Matteo, 27100 Pavia, Italy; s.brugnatelli@smatteo.pv.it; 23 General Surgery Unit, Ospedale del Casentino, 52010 Bibbiena, Italy; andrea.rinnovati@uslsudest.toscana.it; 24 Department of Gastroenterology, Esophageal Surgery Unit, Tuscany Regional Referral Center for the Diagnosis and Treatment of Esophageal Disease, 56126 Pisa, Italy; s.santi@ao-pisa.toscana.it; 25 Department of Medical and Surgical Sciences, University of Bologna, 40138 Bologna, Italy

**Keywords:** preoperative, perioperative, chemotherapy, gastric cancer

## Abstract

Docetaxel associated with oxaliplatin and 5-fluorouracil (FLOT) has been reported as the best perioperative treatment for gastric cancer. However, there is still some debate about the most appropriate number and timing of chemotherapy cycles. In this randomized multicenter phase II study, patients with resectable gastric cancer were staged through laparoscopy and peritoneal lavage cytology, and randomly assigned (1:1) to either four cycles of neoadjuvant chemotherapy (arm A) or two preoperative + two postoperative cycles of docetaxel, oxaliplatin, and capecitabine (DOC) chemotherapy (arm B). The primary endpoint was to assess the percentage of patients receiving all the planned preoperative or perioperative chemotherapeutic cycles. Ninety-one patients were enrolled between September 2010 and August 2016. The treatment was well tolerated in both arms. Thirty-three (71.7%) and 24 (53.3%) patients completed the planned cycles in arms A and B, respectively (*p =* 0.066), reporting an odds ratio for early interruption of treatment of 0.45 (95% confidence interval (CI): 0.18–1.07). Resection was curative in 39 (88.6%) arm A patients and 35 (83.3%) arm B patients. Five-year progression-free survival (PFS) was 51.2% (95% CI: 34.2–65.8) in arm A and 40.3% (95% CI: 28.9–55.2) in arm B (*p =* 0.300). Five-year survival was 58.5% (95% CI: 41.3–72.2) and 53.9% (95% CI: 35.5–69.3) (*p =* 0.883) in arms A and B, respectively. The planned treatment was more frequently completed and was more active, albeit not significantly, in the neoadjuvant arm than in the perioperative group.

## 1. Introduction

Gastric cancer remains one of the major causes of death from cancer [1], despite its declining incidence in recent years. Treatment in Western countries is often administered when the disease is locally advanced, with poor results, and a more promising approach would seem to be preoperative treatment. Two randomized trials on perioperative chemotherapy, one conducted in the United Kingdom [2] and one in France [3], reported an absolute improvement in survival of about 13%. On the basis of these studies, European Society For Medical Oncology (ESMO) guidelines [4] now recommend preoperative treatment for almost all gastric cancer patients.

Recently, the FLOT4-AIO [5] study demonstrated the importance of perioperative therapy in terms of activity, establishing it as a new reference model in this setting. The chemotherapy schedule is the most critical issue. The British and the French trials [2,3] administered three cycles before and three cycles after surgical treatment, while patients in the FLOT4-AIO study underwent four cycles pre-surgery and four post-surgery. However, many of the patients in these studies did not conclude the treatment. Although the preoperative treatment was better accepted, only around 50% of patients were able to undergo treatment after surgery. Yoshikawa [6] and subsequently Aoyama [7] proposed a neoadjuvant treatment with only two rather than four cycles of chemotherapy. We believe that the positive results of these [2,3,5] studies could be related to the preoperative treatment strategy and that four cycles of chemotherapy containing docetaxel, oxaliplatin, and a fluoropyrimidine could be well tolerated by patients.

In 2010, when preoperative chemotherapy still was not a completely acceptable approach in our country, we designed a phase-II randomized trial (GASTRODOC) for resectable gastric cancer to compare the percentage of patients receiving all four planned cycles of neoadjuvant chemotherapy with docetaxel, oxaliplatin, and capecitabine (DOC) with that of patients undergoing the same chemotherapy regimen in a perioperative schedule of two cycles in both the neoadjuvant and adjuvant settings.

## 2. Results

### 2.1. Patients

**Figure 1:**
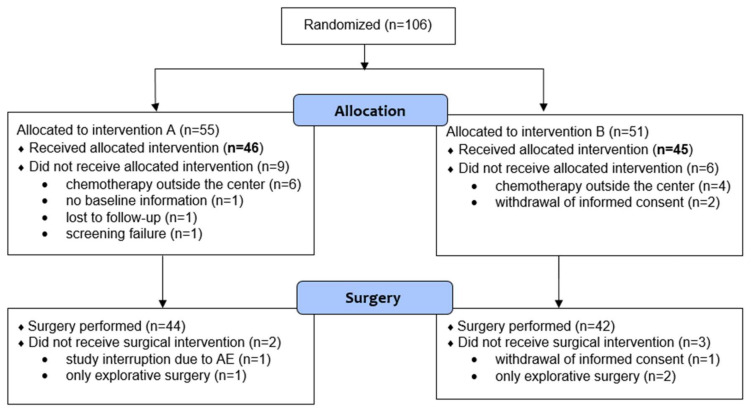
CONSORT diagram for the trial.

Between 21 September 2010 and 19 August 2016, 13 centers enrolled 106 patients (55 in arm A and 51 in arm B). Overall, 91 patients were considered eligible for the analysis (46 in arm A and 45 in arm B). Ten patients (6 in arm A and 4 in arm B) decided to withdraw from the study and underwent chemotherapy elsewhere. Five (3 in arm A and 2 in arm B) other patients were excluded because of lack of data. The trial profile is shown in Figure 1. The baseline characteristics of the study population are shown in Table 1. Patient and disease characteristics were well balanced between the two arms.

Thirty-three (71.7%) and 24 (53.3%) patients completed all the planned cycles in arms A and B, respectively (*p =* 0.066) (Table 2), with a univariate odds ratio (OR) for early termination in arm A with respect to arm B of 0.45 (95% confidence interval (CI): 0.18–1.07). A multivariate logistic regression model, including gender, age, and center, showed a similar OR owing to early treatment termination (OR: 0.49, 95% CI: 0.20–1.18). Unacceptable toxicity and physician’s decision were the most frequent causes of interruption.

The most common non-surgical grade (G)3–G4 adverse events were neutropenia in 8 (17.4%) arm A patients and 4 (8.9%) arm B patients, asthenia in 5 (10.9%) patients in arm A and 3 (6.7%) in arm B patients, and diarrhea in 4 (8.7%) arm A and 4 (8.9%) arm B patients (Table 3).

The percentage of cumulative delivered dose was higher in arm A than in arm B for all the scheduled drugs: cumulative dose of docetaxel was >90% for 25 (54.3%) patients in arm A and 20 (44.4%) patients in arm B; cumulative dose of oxaliplatin was >90% for 31 (67.4%) for arm A and 22 (48.9%) for arm B; cumulative dose of capecitabine was >90% for 22 (47.8%) arm A and 13 (28.9%) for arm B. Doses were reduced in 17 (37.0%) of the 46 arm A patients and 25 (55.6%) of the 45 arm B patients. Of the 91 eligible patients, 22 (24.1%) had at least one dose reduction in the preoperative phase.

Surgical complications, evaluable in 77 (90%) patients, occurred in 23 cases (44 complications). Although the number of patients with complications was fairly similar in the two arms (12 arm A (29.3%) and 11 arm B (30.6%)), the number of complications per group differed substantially (16 in arm A and 28 in arm B). Of note, pneumonia occurred in two cases in arm A versus 7 in arm B, while thoracic-abdominal abscess was observed in no cases in arm A versus 5 in arm B. Three (35%) arm A patients and 7 (66.3%) arm B patients showed more than one complication (Table 4). All surgical complications were resolved with the exception of one arm B patient who died one month post-surgery from intrathoracic/intrabdominal abscess and respiratory failure.

### 2.2. Outcome

We observed one case of complete regression (Becker 1a) in arm A and two in arm B. Arm A patients showed a higher number of subtotal regressions (Becker 1b) (15.9% in arm A versus 7.1% in arm B) and also partial regressions (Becker 2) (38.6% in arm A versus 26.2% in arm B) than arm B. Overall, 86 (94.5%) patients underwent gastric resection; 44 (95.7%) in arm A and 42 (93.3%) in arm B. The reasons for non-resection (or only explorative surgery) during planned surgery were as follows: progression of disease in the surgical bed (one patient) and onset of cerebral ischemia (unrelated to treatment) in arm A (one patient); and consent withdrawal (one patient) and progression of disease in the surgical bed in arm B (2 patients). A total of seven cases of disease progression was observed in the preoperative phase, two in arm A (one of whom underwent subtotal gastrectomy) and five in arm B (three of whom underwent gastrectomy, one total and two subtotal). Palliative surgery was performed in 12 (14%) patients, 5 in arm A, and 7 in arm B, and limited lymphadenectomy was carried out in 4 (4.7%) patients, 2 in each arm. The type of surgical procedure, lymph node dissection, resultant pathological tumor stage, nodal status, and pathological regression are shown in Table 5.

**Figure 2:**
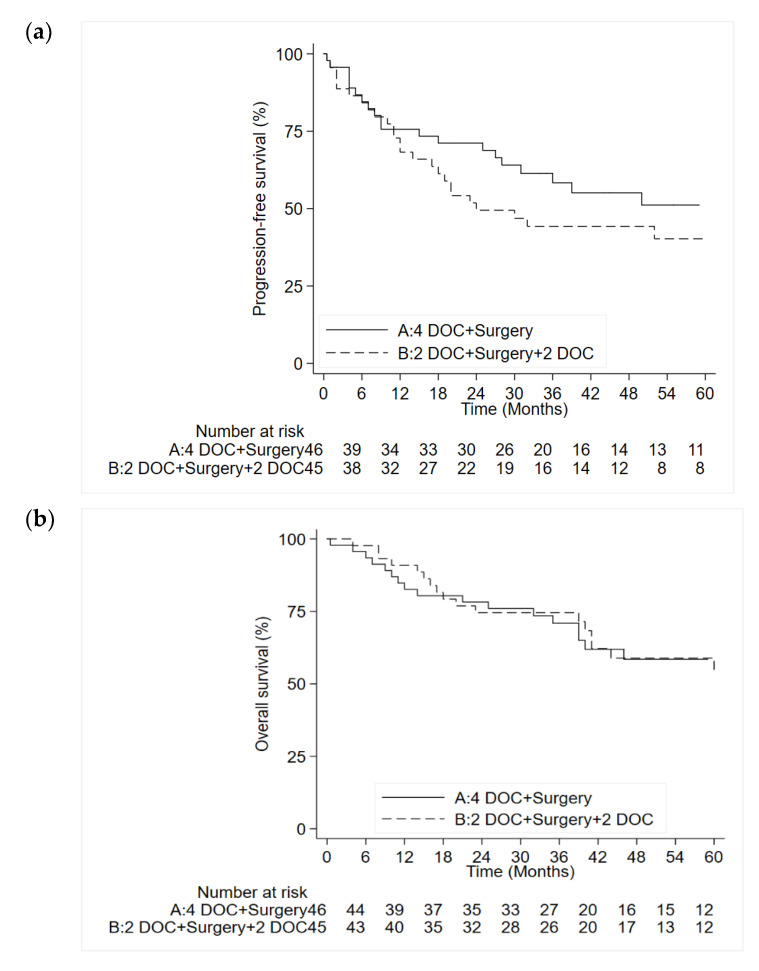
(**a**). Overall survival. (**b**). Progression-free survival. DOC: docetaxel, oxaliplatin, capecitabine.

The median follow-up was 55 months at the time of the analysis (range 4–102). We observed 46 PFS events (21 in arm A and 25 in arm B), 42 cases of disease progression and 4 deaths without evidence of disease progression. Five-year PFS was 51.2% (95% CI: 34.2–65.8) in arm A and 40.3% (95% CI: 28.9–55.2) in arm B (Figure 2a). Median PFS was not reached in arm A and was 24 months (95% CI: 17–not estimable) in arm B (*p =* 0.300). The hazard ratio for the neoadjuvant arm was 0.80 (95% CI: 0.40–1.46) and similar results were obtained when baseline characteristics were included in a multivariate model. Thirty-six patients died during follow-up, 18 (39.1%) in arm A and 18 (40.0%) in arm B. Five-year overall survival was 58.5% (95% CI: 41.3–72.2) and 53.9% (95% CI: 35.5–69.3) (*p =* 0.883) in arms A and B, respectively (Figure 2b). The univariate hazard ratio for arm A was 0.95 (95% CI: 0.81–3.02) and a similar result was obtained when pretreatment covariates were included in the model.

## 3. Discussion

The aim of the present trial was to verify whether neoadjuvant treatment is superior to perioperative therapy in terms of the percentage of completed cycles of chemotherapy in patients with gastric cancer. Our results, albeit not statistically significant, indicate that neoadjuvant treatment (four cycles) was more frequently completed than perioperative treatment (two + two cycles), and several interesting conclusions can be drawn from this. To the best of our knowledge, this is the first study to compare neoadjuvant and perioperative strategies in the multimodal treatment of locally advanced gastric cancer. Given the known low compliance to post-operative chemotherapy schedules, our findings could prove important from a clinical perspective.

Before starting the trial, staging accuracy and surgical techniques were standardized as much as possible by the participating institutes (Istituto Scientifico Romagnolo per lo Studio e la Cura dei Tumori and Italian Research Group for Gastric Cancer (GIRCG) centers), which accounts for the long recruiting period. Laparoscopy was always performed and positive cytology patients were excluded even when carcinosis was not suspected at the diagnostic CT scan. Patients with early gastric cancer (EGC) or cT2 disease, if clinically node-negative, were also excluded from the study in view of their excellent survival rates observed in previous GIRCG studies [8]. The only EGC patients recruited were those with clinically-positive lymph nodes. Patients with carcinoma of the gastric cardia were excluded because of the need for a different neoadjuvant treatment and surgical approach. In brief, the present study focused on non-cardia cT3-cT4 gastric cancer or cT1-cT2 N+ lymph nodes, as described in the GIRCG guidelines [9].

In our study, only 28% of patients in the neoadjuvant arm interrupted the planned four cycles of chemotherapy, the reasons for which include G3–G4 toxicity (which was, however, not as high as that reported in other studies [2,3,5]), consent withdrawal, investigator’s decision, and suspicion of disease progression. In particular, postoperative complications increased the frequency of treatment interruption in arm B, and this aspect should be taken into consideration when planning neoadjuvant or perioperative treatment for gastric cancer patients.

Recently, the FLOT4-AIO (5) phase II–III indicated the feasibility of administering therapy before surgery using either the FLOT (5-FU, folinic acid, oxaliplatin, docetaxel) (93%) or ECF (epirubicin, cisplatin, 5-FU) schedule (92%). However, the difficulty in completing therapy post-surgery for both schedules (47% and 34%, respectively) was confirmed.

In an effort to reduce the negative effects of docetaxel and increase the number of the patients who were able to finish the treatment, we followed the indications of two trials [10,11] and administered the drug on days 1 and 8, rather than in a single dose. This is because a lower daily dosage of docetaxel combined with oxaliplatin and capecitabine has been shown to be less toxic.

With respect to safety profiles, all the observed toxicities were expected and no clinically significant differences were observed between the two arms. Seventy (38 in arm A and 32 in arm B) of the 91 patients undergoing at least one cycle of treatment had at least one G2–G4 complication. This morbidity, related to neoadjuvant treatment, was similar to that reported in the FLOT trial [5].

The number of patients with complications from surgery was similar in both arms and in line with that reported in other studies, whereas the number of post-surgery complications was higher in arm B (28 versus 16 in arm A). Ten of the 23 patients (both arms) experiencing complications from surgery had more than two complications, indicating the potential difficulty in dealing with this subgroup of patients.

Like the FLOT4-AIO [5] and ACCORD [3] trials, the vast majority of our patients underwent R0 gastric resection (88.6% in arm A and 83.3% in arm B). However, in contrast to the aforementioned trials, we did not recruit cT1/T2 N0 patients. Of note, we observed a high number of patients with diffuse type or signet ring cell carcinoma, described in the literature as poorly responsive to neoadjuvant treatments.

The evaluation of the pathological response according to Becker classification was a secondary objective of our trial. In previous studies, complete regression (Becker 1a) ranged between 2% in a phase II trial of paclitaxel/cisplatin [12] and 16% in the FLOT4 trials [5]. In our study, Becker 1a was obtained in three (3.5%) patients. Becker 1b and 2 regression was more frequent in arm A (54.5%) than in arm B (33.3%). Although the pathological response of Becker 1a was lower than that reported in more recent studies, we observed a reduction from cT3-4 (96.5%) to pT3-4 (73.3%) as well as an improvement from cN0 (22%) to pN0 (34%). This downstaging may be attributable to disease regression or a consequence of a staging bias. However, this limitation could have been avoided by excluding early tumors.

The majority of our patients underwent D2 resection or D2 plus lymph node dissection, with a median number of 33 (range 6–88) dissected nodes. Of note, 78% of patients staged as N+ at randomization showed a low median number of metastatic lymph nodes at surgery (median 2, range: 0–26). Although surgeons consider surgery and lymphadenectomy more difficult to perform because of fibrosis in regressed lymphatic stations, morbidity in our patients was not as high as expected.

Considering time-dependent aims, the percentage of patients alive at five years was higher in the neoadjuvant arm than in the perioperative arm, albeit not significantly. These findings merit some comment, the first being that five-year PFS was significantly lower in arm B. Although this did not substantially reduce OS, it will probably worsen over time. The second is that our five-year PFS was 51.2% (95% CI: 34.2–65.8) in arm A and 40.3% (95% CI: 28.9–55.2) in arm B (Figure 2a); median PFS was not reached in arm A and was 24 months (95% CI: 17–not estimable) in arm B. The recent FLOT study reported a median disease-free survival (DFS) of 18 months in the ECF/ECX group and 30 months in the FLOT group. Considering that the current standard of care in Europe is eight perioperative FLOT cycles (four pre- and four post-surgery), our lower number of scheduled cycles obtained survival rates and DFS comparable to those of the German trial. Another important aspect is that, despite the few cases of complete tumor regression (Becker 1a) (probably because small tumors (T1/T2) were not considered in the study), we observed good survival rates that are on a par with those of the FLOT trial [5]. The analysis of the secondary endpoints should be considered exploratory.

A potential limitation of this Italian multicenter trial was the lack of tools available to assess quality of life, which may have helped to explain the failure of patient compliance. Another limitation was the long accrual period, which is a common problem of clinical trials on gastric cancer. The lengthy recruitment may have been owing to the difficulty in proposing this treatment in a period when upfront surgery was the standard treatment for gastric cancer in Italy, and when the importance of close collaboration between clinicians in a multidisciplinary team had still not been acknowledged.

## 4. Patients and Methods

### 4.1. Study Design and Inclusion Criteria

The GASTRODOC study was a randomized, open-label, phase-II trial. Eligible patients were randomly assigned 1:1 to either neoadjuvant (arm A) or perioperative (arm B) chemotherapy. Randomization was performed with a centralized procedure and stratified by center. Treatment was to be started within 8 days of randomization. Investigators and patients were not masked to treatment assignment.

Patients of either gender with advanced (≥T3 or bulky N+), surgically-resectable M0 gastric cancer, excluding cancer of the gastric cardia, were eligible. Other inclusion criteria were age ≥18 and ≤75 years, Eastern Cooperative Oncology Group (ECOG) performance status (PS) 0 or 1, sufficient bone marrow (neutrophils >1.5 × 10^9^/L, platelets >100 × 10^9^/L, hemoglobin >10g/dL), liver, renal function (total bilirubin <1.25 upper limit of normal (UNL), aspartate aminotransferase and alanine aminotransferase <2.5 × UNL, alkaline phosphatase <2.5 × UNL), creatinine <1.5 UNL, and signed written informed consent.

Exclusion criteria included the following: linitis plastica, concurrent chronic systemic immune therapy, clinically-relevant coronary artery disease, and a history of myocardial infarction or of hypertension not controlled by therapy in the previous 12 months. The definition of “bulky lymph node metastases” is in line with the indications of Yoshikawa et al. [13]: “at least one node of ≥3 cm in diameter or at least three consecutive nodes of ≥1.5 cm diameter in first or second level lymph node stations”.

The study was carried out in accordance with Good Clinical Practice and current local legislation, as well as the principles laid down at the 18th World Medical Assembly (Helsinki, 1964), 59th World Medical General Assembly (Seoul, October 2008), Directive 2001/20/EEC of the European Parliament, and other relevant local legislation on not-for-profit studies.

Study management and coordination, as well as remote and onsite monitoring, were carried out at Istituto Scientifico Romagnolo per lo Studio e la Cura dei Tumori (IRST) IRCCS in Meldola, Italy. The cancer centers participating in the trial were required to have their patients operated on by surgeon members of the Italian Research Group for Gastric Cancer (GIRCG). The study protocol was approved by the ethics committee of each participating institute; Istituto Scientifico Romagnolo per lo Studio e la Cura dei Tumori (IRST) IRCCS, Forlì-Meldola, no. 471 of 21 July 2010; Unit of Surgical Oncology, Department of Medicine, Surgery and Neurosciences, University of Siena, Siena, no. E/2012 of 21 October 2020; Medical Oncology and Hematology Unit, Humanitas Cancer Center, Humanitas Clinical and Research Center-IRCCS, Rozzano (Milan), no. 215/12 of 21 September 2012; Santa Maria della Misericordia Hospital, University of Perugia, Perugia, no. 1720 of 16 December 2010; General and Upper GI Surgery Division, University of Verona, Verona, no. 1998 of 11 January 2012; San Giuseppe Hospital, Empoli, no. 905/13 of 17 June 13; Valdarno Hospital, Arezzo, no. 316 of 29 July 2010; Department of Clinical and Experimental Sciences, Surgical Clinic, University of Brescia, Brescia, no. 1776 of 02 September 2014; ASST Bergamo Ovest, Treviglio, no. 45/14 of 26 February 2014; Department of Surgical and Morphological Sciences, University of Insubria, Varese, no. 115/2013 of 17 December 2013; IRCCS San Matteo, Pavia, no. P-2014009265 of 07 April 2014; General Surgery Unit, Ospedale del Casentino, Bibbiena, no. 317 of 29 July 2010; Department of Gastroenterology, Tuscany Regional Referral Center for the Diagnosis and Treatment of Esophageal Disease, Pisa, no. 3544/2012 of 21 June 2012.

The study is registered on the ClinicalTrial.gov website (registration ID: NCT01876927). EudraCT registration number 2010-020189-37.

### 4.2. Staging

The staging system included chest, abdomen, and pelvis computed tomography (CT) scan. Gastroscopy and CT scan of the thorax and abdomen had to be carried out no more than 3 weeks before randomization. A positron emission tomography (PET)/CT scan with 18F-FDG was required before the first treatment cycle. In the event of radiopharmaceutical uptake, the CT/PET scan was to be repeated after the end of the second cycle in arm B, and at the end of the second and fourth cycles in arm A. An electrocardiogram and echocardiogram were carried out 3 weeks before study entry. Optional staging with endoscopic ultrasonography was performed to define the depth of tumor invasion and nodal status. Details regarding criteria for radiological clinical staging are described in GIRCG guidelines [9].

Patients considered fit for the study underwent staging with laparoscopy with peritoneal washing, and those with either positive peritoneal cytology or peritoneal dissemination were subsequently excluded.

### 4.3. Treatment

Treatment was administered over four cycles pre-surgery in arm A, and over two cycles pre-surgery and two cycles post-surgery in arm B, unless progression or unacceptable toxicity occurred or a patient refused treatment and withdrew from the study. Patients underwent surgery 3–6 weeks after the end of the fourth (arm A) or second preoperative (arm B) cycle. Chemotherapy consisted of docetaxel 35 mg/m^2^ on days 1 and 8 in a one-hour infusion, oxaliplatin 80 mg/m^2^ on day 1 in a two-hour infusion, and capecitabine 750 mg/m^2^ twice daily for two weeks. Each cycle was repeated every three weeks. Toxicities were assessed using the National Cancer Institute-Common Toxicity Criteria (NCI-CTC), version 3.0 [14]. A delay in infusion (maximum two weeks) and dose reduction were planned in the event of severe toxicity (see Tables S1–S9 for dose reductions).

Surgery consisted of a complete excision of the tumor with at least a D2 lymphadenectomy, as recommended by JGCA (Japanese Gastric Cancer Association) guidelines [15]. Subtotal gastrectomy was performed when indicated for distal cancer. Splenectomy was carried out only in patients with gastric cancer involving or adhering to the spleen and in those with macroscopic intraoperative involvement of lymph nodes of the splenic hilum or the distal splenic artery.

### 4.4. Assessment and Follow-Up

Data on postoperative complications were recorded in hospital records by the surgical teams according to the Dindo classification [16]. Tumor response after neoadjuvant or perioperative chemotherapy was assessed both clinically and by CT scan or PET/CT scan after the end of the second cycle in arm B, and at the end of the second and fourth cycle in arm A. Tumor regression grade was assessed using the Becker regression criteria [17], which are based on the estimation of the percentage of vital tumor cells in relation to the macroscopically identified tumor bed, and include the following categories: 1a (pathological complete remission), 1b (subtotal regression: <10% residual tumor cells), 2 (partial regression: 10-50% residual tumor cells), and 3 (minor or no regression: >50% residual tumor cells). Pathological staging and resection status (R0, R1, or R2) was performed by each center’s pathologist according to the TNM International Union Against Cancer (UICC) classification, 7th edition [18].

Follow-up started 6 months after randomization and included laboratory exams and clinical visits scheduled every 3 months for the first and second year of randomization, until disease progression. From the third year onwards, follow-up was scheduled for every 6 months. Radiological assessment (abdomen CT and chest CT or X-ray) was performed every 6 months after randomization for a maximum of 5 years, or until disease progression. Gastroscopy was performed one year after randomization and every year thereafter in patients undergoing subtotal gastrectomy, and every 2 years for total gastrectomy, for a maximum of 5 years. The present Clinical Trial adheres to CONSORT guidelines.

### 4.5. Endpoints

The primary endpoint of the GASTRODOC study was the assessment of the percentage of patients receiving all the planned chemotherapeutic cycles in the two arms of treatment (A and B). Secondary endpoints were as follows: safety (number of patients with grade (G) 3–4 of toxicity), proportion of any grade of Becker regression, curative (R0 resection) versus palliative surgery, and evaluation of progression-free survival (PFS) and overall survival (OS). PFS was defined as the time from randomization to the date of the first documented progression (any disease progression) or death from any cause. OS was defined as the time from randomization to the date of death from any cause, or last known alive date.

### 4.6. Statistical Analysis and Sample Size

A total of 90 (45 in each arm) evaluable patients were needed to verify whether arm A was superior to arm B with respect to the percentage of patients completing all the planned chemotherapeutic cycles. Sample size was calculated assuming 80% as the percentage of patients receiving all chemotherapy cycles before surgery (p1), and 50% as the percentage of patients receiving two cycles before and two cycles after surgery (p0), considering alpha = 0.05 (two-sided) and beta = 0.2.

The percentage of patients receiving all the planned chemotherapeutic cycles was calculated and 95% confidence intervals (95% CI) were derived from the exact binominal distribution. The chi-square test was used to determine any significant relationship between treatment arms and interruption of chemotherapy. Univariate and multivariate logistic regression models were carried out to ascertain any potential independent factors associated with treatment interruption. Evaluation of toxicity was carried out by recording main grade G1–G2 and G3–G4 toxicities and dose modifications by arm of treatment. The cumulative dose was defined for each drug as the ratio (%) between cumulative delivered dose and the planned total chemotherapy dose to complete the study treatment. PFS and OS were calculated with the Kaplan–Meier method and the analysis was performed on the eligible population. Hazard ratios were estimated using Cox regression model analysis. Continuous variables are presented as median and range, and qualitative variables are presented as absolute or relative frequencies. *p*-values are based on two-sided testing and the results were considered significant if *p* < 0.05. Statistical analyses were carried out using STATA/MP 15.0 for Windows (Stata Corp. LP, College Station, TX, USA). No correction for multiple testing was applied. No interim analysis was performed.

## 5. Conclusions

In our study, the neoadjuvant approach with four cycles was more frequently completed and more active than the perioperative approach, although the number of patients who completed treatment did not differ significantly between arms. In addition, with regard to the secondary endpoints, all surgical parameters, in particular the pathological response rate according to the Becker classification, suggests the superiority of the neoadjuvant approach over the perioperative one and confirms its safe profile.

## Figures and Tables

**Table 1:** Baseline patient characteristics.

Variable	A: 4 DOC + Surgery (*n =* 46)	B: 2 DOC + Surgery + 2 DOC (*n =* 45)	Overall
Median age, years (range)	63 (39–74)	66 (33–75)	64 (33–75)
Gender			
Male	32 (69.6)	32 (71.1)	64 (70.3)
Female	14 (30.4)	13 (28.9)	27 (29.7)
ECOG performance status			
0	42 (91.3)	43 (95.6)	85 (93.4)
1	4 (8.7)	2 (4.4)	6 (6.6)
cTN			
T2N+	2 (4.3)	1 (2.2)	3 (3.3)
T3N0	8 (17.4)	5 (11.1)	13 (14.2)
T3N+	18 (39.1)	16 (35.6)	34 (37.4)
T4aN0	4 (8.7)	3 (6.7)	7 (7.7)
T4aN+	13 (28.3)	18 (40.0)	31 (34.1)
T4bN+	1 (2.2)	2 (4.4)	3 (3.3)
cT			
T2	2 (4.3)	1 (2.2)	3 (3.3)
T3	26 (56.5)	21 (46.7)	47 (51.6)
T4a	17 (37.0)	21 (46.7)	38 (41.8)
T4b	1 (2.2)	2 (4.4)	3 (3.3)
cN			
N0	12 (26.1)	8 (17.8)	20 (22.0)
N1	14 (30.4)	16 (35.6)	30 (33.0)
N2	4 (8.7)	7 (15.6)	11 (12.0)
N3	3 (6.5)	3 (6.7)	6 (6.6)
N+	13 (28.3)	11 (24.3)	24 (26.4)

ECOG: Eastern Cooperative Oncology Group; DOC: docetaxel, oxaliplatin, capecitabine.

**Table 2:** End of treatment.

End of Treatment	A: 4 DOC + Surgery (*n =* 46)	B: 2 DOC + Surgery + 2 DOC (*n =* 45)	Overall (*n =* 91)	*p*-Value
Chemotherapy according to protocol	33 (71.7)	24 (53.3)	57 (62.6)	0.0668
Interrupted chemotherapy	13 (28.3)	21 (46.7)	34 (37.4)	
Reason for interruption:				
Death	1 (2.2)	1 (2.2)	2 (2.2)	
Investigator’s decision	3 (6.5)	3 (6.7)	7 (7.7)	
Patient withdrew consent	2 (4.3)	3 (6.7)	5 (5.5)	
Progression	1 (2.2)	5 (11.1)	5 (5.5)	
Unacceptable toxicity	5 (10.9)	5 (11.1)	10 (11.0)	
Other	1 (2.2)	4 (8.9)	5 (5.5)	

DOC: docetaxel, oxaliplatin, capecitabine.

**Table 3:** Targeted adverse events reported by patients with at least one cycle of treatment.

Adverse Event	A: 4 DOC + Surgery (*n =* 46) No. of Patients (%)		B: 2 DOC + Surgery + 2 DOC (*n =* 45) No. of Patients (%)	
	G1–G2	G3–G4	G1–G2	G3–G4
Neutropenia	3 (6.5)	8 (17.4)	4 (8.9)	4 (8.9)
Febrile neutropenia	0	0	0	0
Anemia	5 (10.9)	1 (2.2)	6 (13.3)	2 (4.4)
Thrombocytopenia	4 (8.7)	0	1 (2.2)	0
Asthenia	22 (47.8)	5 (10.9)	17 (37.8)	3 (6.7)
Nausea	23 (50.0)	3 (6.5)	22 (48.9)	1 (2.2)
Vomiting	16 (34.8)	2 (4.3)	12 (26.7)	1 (2.2)
Diarrhea	20 (43.5)	4 (8.7)	30 (66.7)	4 (8.9)
Stomatitis	13 (28.3)	2 (4.3)	12 (26.7)	0
Rash	5 (10.9)	0	1 (2.2)	0
Bronchospasm	1 (2.2)	0	1 (2.2)	1 (2.2)
Paresthesia	9 (19.6)	0	9 (20.0)	1 (2.2)
Hand-and-foot syndrome	4 (8.7)	1 (2.2)	3 (6.7)	1 (2.2)
Neurologic toxicity	6 (13.0)	0	1 (2.2)	0
Abdominal pain	9 (19.6)	2 (4.3)	12 (26.7)	1 (2.2)

DOC: docetaxel, oxaliplatin, capecitabine.

**Table 4:** Surgical complications.

Surgical Complication	A: 4 DOC + Surgery	B: 2 DOC + Surgery + 2 DOC	Overall
Patients with evaluation of post-surgery complication	41	36	77
Patients with surgical complication			
Yes	12 (29.3)	11 (30.6)	23 (29.9)
No	29 (70.7)	25 (69.4)	54 (70.1)
Number of surgical complications per patient			
1	9 (75.0)	4 (36.4)	13 (56.5)
2	2 (16.7)	4 (36.4)	6 (26.1)
≥3	1 (8.3)	3 (27.3)	4 (17.4)

DOC: docetaxel, oxaliplatin, capecitabine.

**Table 5:** Characteristics of surgical intervention by treatment arm.

Variable	A: 4 DOC + Surgery	B: 2 DOC + Surgery + 2 DOC	Overall
Evaluable patients	44	42	86
Lauren histotype			
Intestinal	18 (40.9)	23 (54.8)	41 (47.7)
Diffuse	23 (52.3)	14 (33.3)	37 (43.0)
Mixed	2 (4.5)	2 (4.8)	4 (4.7)
Other	0 (0.0)	2 (4.8)	2 (2.3)
Not defined	1 (2.3)	1 (2.4)	2 (2.3)
Type of surgery			
Total gastrectomy	17 (38.6)	18 (42.9)	35 (40.7)
Partial gastrectomy	26 (59.1)	23 (54.8)	49 (57.0)
Other	1 (2.3)	1 (2.3)	2(2.3)
Type of lymphadenectomy			
D1	2 (4.5)	2 (4.8)	4 (4.7)
D2	30 (68.2)	25 (59.5)	55 (64.0)
D2+	12 (27.3)	15 (35.7)	27 (31.3)
Residual tumor classification			
R0	39 (88.6)	35 (83.3)	74 (86.0)
R1	4 (9.1)	7 (16.7)	11 (12.8)
R2	1 (2.3)	0 (0.0)	1 (1.2)
Median no. excised lymph nodes (range)	32 (6–67)	36 (12–88)	33 (6–88)
Median no. metastatic lymph nodes (range)	2 (0–26)	3 (0–21)	2 (0–26)
Becker regression			
1a	1 (2.3)	2 (4.8)	3 (3.5)
1b	7 (15.9)	3 (7.1)	10 (11.6)
2	17 (38.6)	11 (26.2)	28 (32.6)
3	18 (40.9)	25 (59.5)	43 (50.0)
Unknown	1 (2.3)	1 (2.4)	2 (2.3)
Pathological T			
yT0	1 (2.3)	2 (4.8)	3 (3.5)
yT1	5 (11.4)	2 (4.8)	7 (8.1)
yT2	8 (18.2)	5 (11.9)	13 (15.1)
yT3	18 (40.9)	14 (33.3)	32 (37.2)
yT4a	11 (25.0)	16 (38.1)	27 (31.4)
yT4b	1 (2.3)	3 (7.1)	4 (4.7)
Pathological N			
yN0	17 (38.6)	13 (31.0)	30 (34.9)
yN1	7 (15.9)	7 (16.7)	14 (16.3)
yN2	10 (22.7)	9 (21.4)	19 (22.1)
yN3	10 (22.7)	13 (31.0)	23 (26.7)
Tumor localization			
Angulus	2 (4.5)	0 (0.0)	2 (2.3)
Antrum	15 (34.1)	14 (33.3)	29 (33.7)
Corpus	11 (25.0)	13 (31.0)	24 (27.9)
Fundus or cardias	2 (4.5)	4 (9.5)	6 (7.0)
Pylorus	2 (4.5)	2 (4.8)	4 (4.7)
Multiple sites	12 (27.3)	9 (21.4)	21 (24.4)

DOC: docetaxel, oxaliplatin, capecitabine.

## Supplementary Materials

The following are available online at https://www.mdpi.com/2072-6694/12/10/2790/s1, Table S1: Dose reduction for hematological toxicities (oxaliplatin and docetaxel). Table S2: Dose reduction for hematological toxicity (capecitabine). Table S3: Dose reduction for hepatic toxicities (oxaliplatin, docetaxel). Table S4: Dose reduction for hepatic toxicities (capecitabine). Table S5: Dose reduction for renal toxicity. Table S6: Dose reduction for neurologic toxicity. Table S7: dose adjustment for Subsequent treatment cycles. Table S8: Management of anaphylactic reaction. Table S9: Fluid Retention Grading Criteria–Docetaxel.
