# Supplementary material for: Preoperative or Perioperative Docetaxel, Oxaliplatin, and Capecitabine (GASTRODOC Regimen) in Patients with Locally-Advanced Resectable Gastric Cancer: A Randomized Phase-II Trial
Source: Cancers (Basel). 2020 Sep 29;12(10):2790. doi: 10.3390/cancers12102790 (PMC7599648; doi:10.3390/cancers12102790)
Supplement: Supplementary file 1 [file cancers-12-02790-s001.pdf]

cancers

MDPI

Supplementary Materials: Preoperative or Perioperative Docetaxel, Oxaliplatin, and Capecitabine (GASTRODOC Regimen) in Patients with Locally-Advanced Resectable Gastric Cancer: A Randomized Phase-II Trial

Manlio Monti, Paolo Morgagni, Oriana Nanni, Massimo Framarini, Luca Saragoni, Daniele Marrelli, Franco Roviello, Roberto Petrioli, Uberto Fumagalli Romario, Lorenza Rimassa, Silvia Bozzarelli, Annibale Donini, Luigina Graziosi, Verena De Angelis, Giovanni De Manzoni, Maria Bencivenga, Valentina Mengardo, Emilio Parma, Carlo Milandri, Gianni Mura, Alessandra Signorini, Gianluca Baiocchi, Sarah Molfino, Giovanni Sgroi, Francesca Steccanella, Stefano Rausei, Ilaria Proserpio, Jacopo Viganò, Silvia Brugnatelli, Andrea Rinnovati, Stefano Santi, Giorgio Ercolani, Flavia Foca, Linda Valmorri, Dino Amadori, and Giovanni Luca Frassineti

Dose modification of study medications

Dose reduction is planned in case of any type of severe toxicity, hematological and non-hematological. Dose adjustments are to be made on the basis of the greatest degree of toxicity. Toxicities are graded using NCI common toxicity criteria grading.

Doses that have been reduced due to toxicity cannot be re-escalated.

Hematogical toxicities

Table S1. Dose reduction for hematological toxicities (oxaliplatin and docetaxel).

During a course of therapy and/or before each infusion	Action to be taken at each infusion
ANC ≥ 0.5 but < 1.5 × 10^9 /L	**Delay [3] infusion until recovery to** ANC ≥ 1.5 × 10^9/L<br>**No dose reduction is required**
ANC < 0.5 × 10^9/L **or**<br>Febrile neutropenia [1] **or**<br>Neutropenic infection [2] **or**<br>second appearance of ANC<1.0 x 10^9 /L	**Delay [3] infusion until recovery to** ANC ≥ 1.5 × 10^9/L<br>**and ↓ dose (oxaliplatin and docetaxel) by 20%**
Platelets ≥ 25.0 but < 100.0 × 10^9/L	**Dose delay [3] until recovery to ≥ 100 × 10^9/L**
Platelets < 25.0 × 10^9/L	**Delay [3] infusion until** ≥ 100.0 × 10^9/L **and ↓ dose (oxaliplatin and docetaxel) by 20%**

[1] Febrile neutropenia (grade 4 neutropenia and concomitant fever), single oral T° ≥ 38.5°C or 3 elevations to ≥ 38.1°C during a 24-hour period; [2] Neutropenic infection grade 4 neutropenia and concomitant infection ≥ grade 2 (documented clinically or by laboratory tests); [3] Delay (for a maximum of 2 weeks) until counts reach lower limits for treatment.

If grade-4 myelotoxicity persists after 2 weeks' delay, treatment must be interrupted. In the presence of symptoms possibly related to hematological toxicity (bleeding, infections), a complete blood count (CBC) must be performed immediately and the treatment dose reduced. The next cycle can be administered only when values have recovered.

Table S2. Dose reduction for hematological toxicity (capecitabine). The daily dose of capecitabine is reduced by 25% at the first occurrence of grade-3 toxicity or the second occurrence of grade-2 toxicity. The daily dose is reduced by 50% at the first occurrence of grade-4 toxicity, the second occurrence of grade-3 toxicity and the third occurrence of grade-2 toxicity. Capecitabine is discontinued at the second occurrence of grade 4 toxicity.

Grades of Toxicity	Action to Be Taken during Toxicity	Dose Reduction for the Next Cycle
Grade 1	Delay[3] oral administration until recovery to grade 0	No dose reduction
Grade 2		
- First event	Delay[3] oral administration until recovery to grade 0	No dose reduction
- Second event	Delay[3] oral administration until recovery to grade 0	Dose reduction by 25%
- Third event	Delay[3] oral administration until recovery to grade 0	Dose reduction by 50%
- Fourth event	Interrupt drug therapy	Not applicable
Grade 3		
- First event	Delay[3] oral administration until recovery to grade 0	Dose reduction by 25%
- Second event	Delay[3] oral administration until recovery to grade 0	Dose reduction by 50%
- Third event	Interrupt drug therapy	Not applicable
Grade 4		
- First event	Interrupt drug therapy (if the toxicity is considered drug-specific) or delay[3] treatment until recovery to grade 0	Dose reduction by 50%
- Second event	Interrupt drug therapy	Not applicable

Preventive growth factors (G-CSF or GM-CSF) are not recommended. Their use may be considered in cases of prolonged or complicated severe neutropenia.

Non hematological toxicities

The dose modification grid for drug medications is as follows:

Hepatic toxicity

Liver function tests will be carried out before each planned treatment.

Table S3. Dose reduction for hepatic toxicities (oxaliplatin, docetaxel).

Serum liver function tests on day 1					
**SGOT (AST) × UNL and or SGPT (ALT) × UNL**	**Alkaline phosphatase × UNL**		**Total bilirubin**		**Chemotherapeutic drugs (oxaliplatin, docetaxel)**
≤ 2.0	and	≤ 5.0	and	WNL	No change
> 2.0 - ≤ 5.0	and	≤ 2.5	and	WNL	No change
> 2.0 - ≤ 5.0	and	> 2.5 - ≤ 5.0	and	WNL	Dose reduction by 20% [1]
> 5.0	or	> 5.0	or	UNL	Delay and then reduction by 20% [2]

[1] If liver toxicity worsens (further elevation of any liver function test) after dose reduction, patient should go off treatment; [2] Delay (for a maximum of 2 weeks) until liver function tests reach lower values acceptable for treatment.

If chemotherapy must be delayed due to liver toxicity, liver function tests should be performed weekly until liver function tests reach lower limits acceptable for treatment.

Table S4. Dose reduction for hepatic toxicities (capecitabine).

Grades of toxicity	Action to be taken during toxicity	Dose reduction for the next cycle
Grade 1	Delay[3] oral administration until recovery to grade 0	No dose reduction
Grade 2		
- First event	Delay[3] oral administration until recovery to grade 0	No dose reduction
- Second event	Delay[3] oral administration until recovery to grade 0	Dose reduction by 25%
- Third event	Delay[3] oral administration until recovery to grade 0	Dose reduction by 50%
- Fourth event	Interrupt drug therapy	Not applicale
Grade 3		
- First event	Delay[3] oral administration until recovery to grade 0	Dose reduction by 25%
- Second event	Delay[3] oral administration until recovery to grade 0	Dose reduction by 50%
- Third event	Interrupt drug therapy	Not applicable
Grade 4		
- First event	Interrupt drug therapy (if the toxicity is considered drug-specific) or delay[3] treatment until recovery to grade 0	Dose reduction by 50%
- Second event	Interrupt drug therapy	Not applicable

Renal toxicity

Table S5. Dose reduction for renal toxicity.

Serum Creatinine mg/dL		Creatinine Clearance ml/min	Chemotherapeutic dose administration
≤ 1.5	and	any	No change
> 1.5 - ≤ 2.0	and	≥ 50	Delay
> 2.0	and	any	Delay

Capecitabine is controindicated if creatinine clearance is < 29 mL/min, while capecitabine can be reduced by 25% if creatinine clearance is 30–50 mL/min.

If renal criteria for treatment are not met on day 1 of the chemotherapy cycle, re-evaluate weekly. If renal criteria for treatment are not met after 2 weeks, **patients should go off treatment.**

Neurologic toxicity

Table S6. Dose reduction for neurologic toxicity.

Grade of neurologic toxicity at the time of planned treatment	Docetaxel and oxaliplatin doses to be admnistered
0 or 1	No change
2 / 3	Delay oxaliplatin and docetaxel treatment doses by 1 week, if grade ≥ 2 persists for > 2 weeks patient should go off study.<br>If patient recovers to grade 1 toxicity, then dose of both drugs is reduced by 20%.<br>If there is no recovery to grade 1 in two weeks, discontinue treatment.
4	**Patient is discontinued from the study**

If neurologic toxicity is grade > 1 at the time of the planned treatment, treatment is not administered. The patient is tested and monitored weekly. If toxicity has not resolved to grade ≤ 1 after two weeks, the patient must go off treatment.

Diarrhea and stomatitis and hand-foot sindrome.

Capecitabine administration will be interrupted if patient develops grade ≥ 2 diarrhea, stomatitis or hand-foot sindrome, and will be resumed after resolution to grade ≤ 1. Subsequent treatment cycles will be given at the appropriate dose adjustment (table S7). If toxicity reappears or recovery is not complete after a 2-week delay despite these reductions, treatment will be interrupted.

Table S7. Dose adjustment for subsequent treatment cycles (capecitabine).

Grades of toxicity	Action to be taken during the toxicity	Dose reduction for the next cycle
Grade 1	Delay[3] oral administration until recovery to grade 0	No dose reduction
Grade 2		
- First event	Delay[3] oral administration until recovery to grade 0	No dose reduction
- Second event	Delay[3] oral administration until recovery to grade 0	Dose reduction by 25%
- Third event	Delay[3] oral administration until recovery to grade 0	Dose reduction by 50%
- Fourth event	Interrupt drug therapy	Not applicable
Grade 3		
- First event	Delay[3] oral administration until recovery to grade 0	Dose reduction by 25%
- Second event	Delay[3] oral administration until recovery to grade 0	Dose reduction by 50%
- Third event	Interrupt drug therapy	Not applicable
Grade 4		
- First event	Interrupt drug therapy (if the toxicity is considered drug-specific) or delay[3] treatment until recovery to grade 0	Dose reduction by 50%
- Second event	Interrupt drug therapy	Not applicable

Anaphylactic reactions

Docetaxel can induce an anaphylactic reaction. It will therefore be helpful to administer steroids and antihistamines before each docetaxel infusion in patients who experience this reaction. Description and suggested management of docetaxel hypersensitivity reaction is as follows:

Table S8. Management of anaphylactic reaction.

**Mild symptoms: localized cutaneous reaction such as mild pruritus, flushing, rash**	• **consider decreasing the rate of infusion until recovery of symptoms, stay in bed, then, complete docetaxel infusion at the initial planned rate**
Moderate symptoms: any symptom not listed above (mild symptoms) or below (severe symptoms), such as generalized pruritus, flushing, rash, dyspnea, hypotension with systolic blood pressure (BP) > 80 mm Hg	• stop docetaxel infusion<br>• **give IV metilprednisolone 125 mg (1-2 fl) and/or clorfenamina 10 mg IV,**<br>resume docetaxel infusion after recovery of symptoms
Severe symptoms: bronchospasm, generalized urticaria, systolic BP < 80 mm Hg, angioedema	• stop docetaxel infusion<br>• **give IV clorfenamina 10 mg and/or metilprednisolone 125 mg (1-2 fl) and/or epinephrine as needed**<br>• Whenever possible resume docetaxel infusion within 3 hours of recovery or reinfuse the patient within 72 hours using dexamethasone 10 mg IV and/or **clorfenamina 10 mg** IV 30 minutes before resuming infusion.<br>• If severe reaction recurs despite additional premedication, the **patient will go off treatment**
Anaphylaxis (NCI Grade 4 reaction)	• **no further treatment**

Management of Subsequent Treatment Cycles: The recommended pretreatment for subsequent infusions is clorfenamina 10 mg IV and 10 mg dexamethasone IV 60 minutes prior to docetaxel infusion. This is in addition to the prescribed oral dexamethasone.

Patients with hypersensitivity reactions to docetaxel are at risk of recurrent reactions. For patients who experience moderate or severe hypersensitivity reactions, docetaxel should be administered over a period of 2 hours for subsequent treatment courses in addition to premedication as outlined above.

These patients must be informed of the potential risk of recurrent allergic reactions and carefully monitored.

If the initial reaction is grade 4 according to NCI Common Toxicity Criteria for Allergy, the patient will receive no further treatment and will **go off treatment**. If a second severe reaction (grade 3) occurs despite additional premedications as outlined above, **the patient will go off treatment.** In the event of late hypersensitivity symptoms, e.g. appearance of localized or generalized pruritus within 1 week of treatment, symptomatic treatment may be given (e.g. oral antihistamine). Additional oral or IV premedication with antihistamine may also be given for the next cycle of treatment depending on the intensity of the reaction observed. No dose reduction will be made for any patient.

Fluid Retention

No docetaxel dose reduction is planned for fluid retention syndrome. For the purpose of toxicity evaluation, "fluid retention" is defined as the development of trace edema or cytologically negative pleural effusion, ascites, or pericardial effusion, and is graded as "mild", "moderate" or "severe" according to the definitions in the table below. Investigators should report the highest grade of edema or effusion.

Table 9. Fluid retention grading criteria (docetaxel).

EDEMA	SEVERITY GRADING	ACTION
Asymptomatic	MILD<br>Grade 1	Asymptomatic,<br>No intervention required
Symptomatic	MODERATE<br>Grade 2	Symptomatic,<br>Intervention may be required
Symptomatic, resulting in drug discontinuation.	SEVERE<br>Grade 3	Symptomatic,<br>Intervention urgently required

Patients developing new onset or symptomatic edema, or other signs of increasing fluid retention, should be treated with oral diuretics. Regimens which have been found to be effective in the management of fluid retention due to docetaxel are listed below. Diuretic therapy may be initiated in the order listed at the investigator's discretion: Spironolactone 50 mg daily up to TID.

Furosemide 40 mg PO daily if not responsive to spironolactone. Potassium supplements may be given as needed.

If, after a trial of 2 weeks the treatment is ineffective, treat with furosemide 20 mg PO daily plus metolazone 2.5 mg PO daily with potassium supplementation as needed.

Further therapy should be personalized, depending on the clinical situation. The clinical tolerance of the patients, the overall tumour response and the medical judgment of the investigator will determine if it is in the patient's best interest to continue or discontinue treatment.

If severe fluid retention is not responsive to symptomatic therapy, the patient should go off treatment.

Other Toxicities Not Specified Above

In case of toxicities ≤ grade 2, manage symptomatically if possible and resume treatment without dose reduction.

In case of toxicities ≥ grade 3, the drug suspected to be responsible for the toxicity should be delayed (for a maximum of 2 weeks) until resolution to ≤ grade 1 and then reduced by 20%. In case of no recovery within 2 weeks' delay or recurrence of the same toxicity, treatment should be interrupted.

No further dose increases are allowed.

Alopecia (any grade) is excluded from these recommendations.

In case of unstable angor related to 5-FU administration, or myocardial infarction, treatment will be stopped.

Management of specific toxicities related to Capecitabine

Expected adverse effects of treatment: delayed diarrhea.

Delayed diarrhea

Treatment with Capecitabine may induce delayed diarrhea.

No prophylactic agent will be given; in particular, loperamide will not be prescribed for prophylaxis. Patients will be advised to stop any laxative treatment they are taking and to avoid food and beverages that could accelerate intestinal transit.

Curative treatment

As soon as the first liquid stool or abnormal bowel movement occurs, the patient must immediately start loperamide, 2 capsules p.o., then 1 capsule p.o. every 2 hours for at least 12 hours and up to 12 hours of the last liquid stool, without exceeding a total treatment duration of 48 hours. Oral rehydration with large volumes of water and electrolytes will be prescribed during the whole episode.

If diarrhea persists for longer than 48 hours despite the recommended loperamide treatment, a 7-day oral prophylactic broad-spectrum antibiotic therapy with fluoroquinolone will be started after medical advice. The patient may require hospitalization for parenteral support. Loperamide will be replaced by another antidiarrheal treatment (e.g. octreotide).

Oral fluoroquinolone should be given to patients with:

- any grade-4 diarrhea;
- grade-3 diarrhea plus neutropenia, or in the event of fever.

Patients who experience concomitant vomiting or fever, or those with a performance status > 2, should be hospitalized immediately for rehydration.

© 2020 by the authors. Licensee MDPI, Basel, Switzerland. This article is an open access article distributed under the terms and conditions of the Creative Commons Attribution (CC BY) license (http://creativecommons.org/licenses/by/4.0/).
